# *Plasminogen Activator Inhibitor-1* 4G/5G Gene Polymorphism and Coronary Artery Disease in the Chinese Han Population: A Meta-Analysis

**DOI:** 10.1371/journal.pone.0033511

**Published:** 2012-04-04

**Authors:** Yan-yan Li

**Affiliations:** Department of Geriatrics, First Affiliated Hospital of Nanjing Medical University, Nanjing, China; University of Tor Vergata, Italy

## Abstract

**Background:**

The polymorphism of *plasminogen activator inhibitor-1* (*PAI-1*) 4G/5G gene has been indicated to be correlated with coronary artery disease (CAD) susceptibility, but study results are still debatable.

**Objective and Methods:**

The present meta-analysis was performed to investigate the association between *PAI-1* 4G/5G gene polymorphism and CAD in the Chinese Han population. A total of 879 CAD patients and 628 controls from eight separate studies were involved. The pooled odds ratio (OR) for the distribution of the 4G allele frequency of *PAI-1* 4G/5G gene and its corresponding 95% confidence interval (CI) was assessed by the random effect model.

**Results:**

The distribution of the 4 G allele frequency was 0.61 for the CAD group and 0.51 for the control group. The association between *PAI-1* 4G/5G gene polymorphism and CAD in the Chinese Han population was significant under an allelic genetic model (OR = 1.70, 95% CI = 1.18 to 2.44, *P* = 0.004). The heterogeneity test was also significant (*P*<0.0001). Meta-regression was performed to explore the heterogeneity source. Among the confounding factors, the heterogeneity could be explained by the publication year (*P* = 0.017), study region (*P* = 0.014), control group sample size (*P* = 0.011), total sample size (*P* = 0.011), and ratio of the case to the control group sample size (RR) (*P* = 0.019). In a stratified analysis by the total sample size, significantly increased risk was only detected in subgroup 2 under an allelic genetic model (OR = 1.93, 95% CI = 1.09 to 3.35, *P* = 0.02).

**Conclusions:**

In the Chinese Han population, PAI-1 4G/5G gene polymorphism was implied to be associated with increased CAD risk. Carriers of the 4G allele of the *PAI-1* 4G/5G gene might predispose to CAD.

## Introduction

The plasminogen activator inhibitor-1 (PAI-1), a key regulating factor, determines endogenous fibrinolysis activity. The elevated plasma concentration or activity of PAI-1 is one of the relevant signs of atherosclerosis and thrombus disease [1]. Many studies have demonstrated that elevated PAI-1 activity is an independent predictor of coronary artery disease (CAD) and myocardial infarction (MI) [2]. Local plasmin generation is inhibited by elevated PAI-1, leading to decreased fibrinolysis activity, thrombosis, and fibrin deposition; local plasmin generation likewise facilitates atherosclerosis progress and vascular occlusion.

The *PAI-1* gene, located in 7q21.3–22, spans 12.3 kb and contains 9 exons and 8 introns. The polymorphism of the 4G/5G gene is located in the *PAI-1* gene promoter region. The 5^th^ guanine (G base) is inserted or deleted in the 4 G sequence in the –675^th^ base of the transcription initial point upstream. The *PAI-1* gene has three genotypes, namely, 4G4G, 4G5G, and 5G5G. 4G4G allele carriers always have higher plasma PAI-1 activity than 4G5G and 5G5G carriers [3].

**Table 1:** Characteristics of the investigated studies of the association between *PAI-1* 4G/5G gene polymorphism and coronary artery disease.

Author	Year	Region	CAD			Control			Sample Size (CAD/control)
			4G4G	4G5G	5G5G	4G4G	4G5G	5G5G	
Dai Y[9]	2001	Beijing	85	110	55	35	48	12	250/95
Fu Y [11]	2001	Beijing	58	49	16	38	85	49	123/172
Guan LX [12]	2002	Shandong	50	52	24	23	70	28	126/121
Li XS[10]	2002	Guangdong	13	18	5	5	11	0	36/16
Wang YS[13]	2003	Shandong	8	35	24	2	7	21	67/30
Yin SQ[14]	2003	Tianjin	24	22	9	11	29	8	55/48
Xia DS[15]	2006	Tianjin	79	67	20	18	28	17	166/63
Zhan M [16]	2003	Tianjin	40	14	2	25	52	6	56/83

Abbreviations: PAI-1: plasminogen activator inhibitor-1.

In 2006, Ye et al. performed a meta-analysis on seven hemostatic gene polymorphisms in coronary disease in Caucasians and African-Americans. They reported that the *PAI-1* (–675) 4G variant yields a per-allele relative risk of 1.06 (1.02 to 1.10) for coronary disease. However, their study had an indication of publication bias [4]. In 2009, Isordia-Salas I et al. found that the 4G allele is an independent risk factor for acute MI in young patients, similar to smoking, hypertension, and a family history of inherited cardiovascular disease in Mexico [5]. In 2010, Abboud N et al. found that the MI risk is notably high in 4 G carriers with elevated plasma PAI-1 in a Tunisian population [6]. However, in 2011, Rallidis LS et al. found that the 4 G allele of PAI-1 4G/5G polymorphism is less frequent among very young survivors of MI compared with that in matched controls in Greece [7]. In 2011, Ashavaid TF et al. found that *PAI-1* 4G/5G gene polymorphism does not affect the severity of CAD in an Indian population [8].

Studies on the association between *PAI-1* 4G/5G gene polymorphism and CAD have been extensively performed in China, but the results are still disputable. In 2001, Dai Y et al. reported that *PAI-1* 4G/5G gene polymorphism is not associated with susceptibility for CAD [9]. The point was favored by another study [10]. However, other studies had opposite observations [11]–[16]. Therefore, the present meta-analysis, which included 1,507 participants, was performed to draw a valuable conclusion on the relationship between *PAI-1* 4G/5G gene polymorphism and CAD in the Chinese Han population (File S1).

**Table 2:** Summary of meta-analysis of association of *PAI-1* 4G/5G gene polymorphism and coronary artery disease risk.

	Number	CAD size	Con size	Pooled OR (95% CI)	P value	P_heterogeneity_
Chinese Han population	8	879	628	1.70(1.18–2.44)	0.004*	<0.0001*
Subgroup 1(total size>200)	4	665	451	1.56(0.95–2.57)	0.08	0.04*
Subgroup 2(total size<200)	4	214	177	1.93(1.09–3.35)	0.02*	<0.0001*

Abbreviations: CI: confidence interval; OR: odds ratio; number: literature number; CAD size: the total number of CAD cases; Con size: control group size, the total number of control group;

*P<0.05.

## Materials and Methods

### Publication Search and Inclusion Criteria

The electronic databases PubMed, Embase, Web of Science, China Biological Medicine Database, and China National Knowledge Infrastructure were searched using the MeSH terms “coronary artery disease” or “coronary heart disease,” “polymorphism,” “*plasminogen activator inhibitor-1*,” “gene,” and “Chinese.” The included studies were published from 2001 to 2008 (last research updated on July 04, 2011).

The selected studies had to be in accordance with the following major criteria: a) evaluation of *PAI-1* 4G/5G gene polymorphism and CAD in a Chinese Han population; b) diagnosis of CAD in accordance with the examination results of coronary arteriography (>50% stenosis of at least one major vessel), as well as clinical symptoms combined with electrocardiogram, echocardiography, treadmill exercise test, and myocardial perfusion imaging in emission computed tomography (ECT); c) controls proven to have no CAD either by coronary arteriography or by medical history, blood test, physical examination, electrocardiogram, echocardiography, treadmill exercise test, and ECT; and d) no other cardiovascular risk factors such as hypertension, hyperlipidemia, and diabetes mellitus (applied to all participants, including cases and controls).

**Figure 1:**
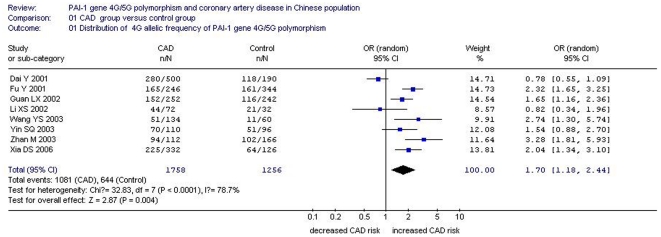
Forest plot of coronary artery disease associated with 4G/5G gene polymorphism (distribution of 4G allelic frequency of *plasminogen activator inhibitor-1* 4G/5G gene).

### Data Extraction

The data were obtained according to a standard protocol. Studies that were repeated, did not meet the inclusion criteria, or provided little data were excluded. If the same data appeared in different works, the result was only used once. The collected data comprised the name of the first author, publication year, region, number of genotypes, genotyping, study design, matching criteria, and total number of cases and controls.

**Table 3:** The confounding factors for the potential sources of heterogeneity studied by meta-regression.

Study	Year	Region	Case size	Con size	Tot size	RR	Weight	OR	LnOR
Dai Y [9]	2001	1	250	95	345	2.63	14.71	0.78	–0.25
Fu Y [11]	2001	1	123	172	295	0.72	14.73	2.32	0.84
Guan LX [12]	2002	1	126	121	247	1.04	14.54	1.65	0.50
Li XS [10]	2002	2	36	16	52	2.25	8.57	0.82	–0.20
Wang YS [13]	2003	1	67	30	97	2.23	9.91	2.74	1.01
Yin SQ [14]	2003	1	55	48	103	1.15	12.08	1.54	0.43
Xia DS [15]	2006	1	166	63	229	2.63	13.81	3.28	1.19
Zhan M [16]	2003	1	56	83	139	0.67	11.64	2.04	0.71

Abbreviations: Year: publication year; Region 1: northern China; Region 2: southern China; Case size: CAD group sample size; Con size: the total number of control group; Tot size: total sample size; RR: the ratio of case size to control size; LnOR: the natural logarithm of odds ratio for the 4G allelic distribution frequency of *PAI-1* gene 4G/5G polymorphism between CAD and control groups.

### Statistical Analysis

The association between *PAI-1* 4G/5G gene polymorphism and CAD was compared using the odds ratio (OR) corresponding to a 95% confidence interval (CI). The presence of inter-study heterogeneity was calculated by the Chi-square-based Q-test, and significance was set at the *P*<0.05 level [17]. The inconsistency index *I* ^2^ was calculated to assess the variation caused by the heterogeneity. If heterogeneity was observed among the studies, the random-effects model was used to estimate the pooled OR (the DerSimonian and Laird method) [18]. Otherwise, the fixed-effects model was adopted (the Mantel–Haenszel method) [19]. The Z test was used to determine the pooled OR with the significance set at *P*<0.05.

Fisher’s exact test was used to assess the Hardy–Weinberg equilibrium (HWE) with the significance set at *P*<0.05. The potential publication bias was estimated by the funnel plot. Egger’s linear regression test on the natural logarithm scale of the OR was used to assess the funnel plot asymmetry; the significance was set at the *P*<0.05 level [20]. STATA 10.0 software was used to perform all statistical analyses (StataCorp, College Station, TX, USA).

**Table 4:** The meta-regression results among 8 studies.

	Coefficient	Standard error	T value	P value	95% Confidence interval
RR	0.8320814	0.114808	7.25	0.019*	0.3381026∼1.32606
Control size	0.0273875	0.0028546	9.59	0.011*	0.015105∼0.03967
Region	−1.174875	0.1413874	−8.31	0.014*	−1.783216∼−0.5665344
Publication year	0.1874112	0.0250733	7.47	0.017*	0.0795295∼0.2952929
Total size	−0.0120356	0.0012977	−9.27	0.011*	−0.0176192∼-0.0064519
Cons	−374.732	50.28602	−7.45	0.018*	−591.0953∼−158.3687

Abbreviations: Control size: Control group sample size; Total size: Total sample size; RR: the ratio of case size to control size; Coefficient: regression coefficient;The regression coefficients are the estimated increase in the lnOR per unit increase in the covariate as control size, RR, total size, region and publication year; cons:constant item.

* P<0.05.

## Results

### Studies and Populations

A total of 22 papers were obtained by the literature search, among which 8 fit the inclusion criteria. Of the 14 excluded studies, 3 papers were redundant studies, 5 were reviews, 2 deviated from the HWE, and 4 were not involved with *PAI-1* 4G/5G gene polymorphism. The data were collected from 879 CAD patients and 628 controls (Table 1, File S2) [9]–[10], [11]–[16]. The five surveyed regions comprised the provinces of Beijing, Shandong, Guangdong, Jiangsu, and Tianjin.

**Figure 2:**
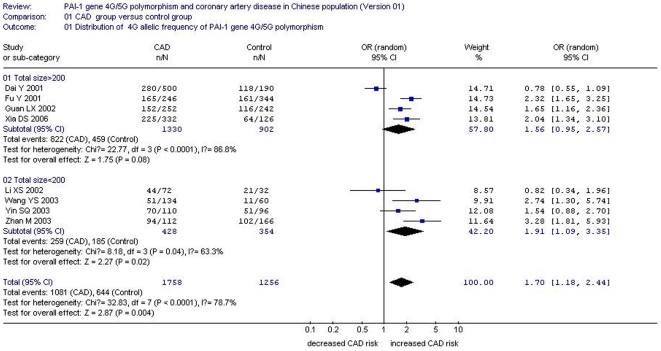
Forest plot of coronary artery disease associated with 4G/5G gene polymorphism (distribution of 4G allelic frequency of *plasminogen activator inhibitor-1* 4G/5G gene) stratified by total size.

### Pooled Analyses

The distribution of the 4 G allele frequency was 0.61 for the CAD group and 0.51 for the control group. The association between *PAI-1* 4G/5G gene polymorphism and CAD in the Chinese Han population was significant under an allelic genetic model (OR = 1. 70, 95% CI = 1.18 to 2.44, *P* = 0.004) (Table 2 and Figure 1).

The heterogeneity test was also significant (*P*<0.0001). Meta-regression was performed to explore the heterogeneity source. Among the confounding factors, the heterogeneity could be explained by the publication year (*P* = 0.017), study region (*P* = 0.014), control group sample size (*P* = 0.011), total sample size (*P* = 0.011), and RR (*P* = 0.019). In a stratified analysis by the total sample size, significantly increased CAD risk was detected in subgroup 2 under an allelic genetic model (OR = 1.93, 95% CI = 1.09 to 3.35, *P* = 0.02). No significant increased CAD risk was detected in subgroup 1 under an allelic genetic model (OR = 1.56, 95% CI = 0.95 to 2.57, *P* = 0.08). Studies with a total size of >200 were included in subgroup 1, and the residual studies with a total size of <200 were included in subgroup 2 (Tables 2, 3, and 4 and Figure 2).

**Figure 3:**
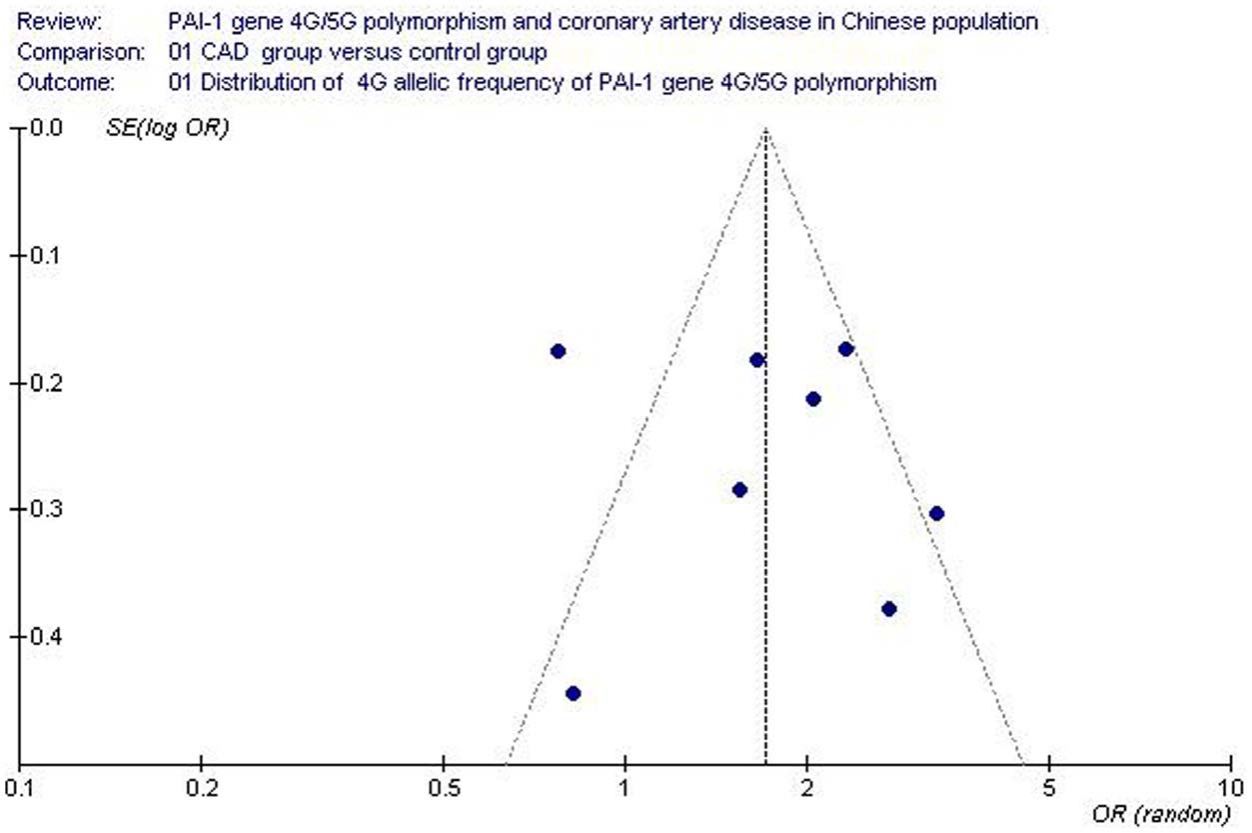
Funnel plot for studies of the association of coronary artery disease and *plasminogen activator inhibitor-1* 4G/5G gene polymorphism (distribution of 4 G allelic frequency of *plasminogen activator inhibitor-1* 4G/5G gene). The horizontal and vertical axis correspond to the OR and confidence limits. OR: odds ratio; SE: standard error.

### Bias Diagnostics

The publication bias of the studies was assessed using the funnel plot and Egger’s test. Publication bias was not seen in the funnel plot (Figure 3). No statistically significant difference was found in the Egger’s test, indicating low publication bias in the current meta-analysis (*T* = 2.09, *P* = 0.082).

## Discussion

The current meta-analysis included 879 CAD patients and 628****controls. Significant association was observed between *PAI-1* 4G/5G gene polymorphism and CAD under an allelic genetic model in the Chinese Han population (OR = 1.70). This finding suggested that the carriers with the 4****G allele of the *PAI-1* 4G/5G gene in the Chinese Han population might be predisposed to CAD.

In the subsequent meta-regression, the confounding factors (publication year, study region, control size, total size, and RR) could explain the heterogeneity. This result suggested that the non-uniformity in the abovementioned factors contributed to the heterogeneity among the individual studies. In a stratified analysis by the total size, significantly increased risk was only detected in subgroup 2 under an allelic genetic model. After adjusting for the total sample size, unexplained heterogeneity still existed. The association of *PAI-1* 4G/5G gene polymorphism with CAD was evidently weakened in subgroup 1 and not yet significant (OR = 1.56). In contrast, the association of *PAI-1* 4G/5G gene polymorphism with CAD was evidently strengthened in subgroup 2 (OR = 2.03). Hence, further studies that can provide a match between the sample sizes of cases and controls are warranted.

PAI-1 is a glycoprotein that belongs to the serine protease inhibitor superfamily. It is equimolecularly combined with the tissue plasminogen activator (tPA) single chain, double chains, and double chain urokinase plasminogen activator (uPA). Consequently, tPA and uPA activities are rapidly inhibited by PAI-1. PAI-1 is also an essential regulatory substance for in vivo plasminogen activation. PAI-1, which is synthesized by endothelial cells and the liver, is regulated by hormones and neurotransmitters. A dose-response relationship has been found between PAI-1 and such metabolism syndrome-related factors as obesity, hypertriglyceridemia, low high-density lipoprotein hyperlipidemia, hyperglycemia, hyperinsulinemia, and physical labor deficiency [21].

*PAI-1* 4G/5G gene polymorphism is one of the DNA sequence variations that plays a key role in regulating *PAI-1* gene expression. Studies have shown that the PAI-1 activity of the 4****G allele promoter is higher than that of 5****G in a cytokine-stimulated state. *PAI-1* 4G/5G gene polymorphism also influences *PAI-1* gene transcription similarly in non-stimulated cells [22]. The 4****G or 5****G allele could be combined with protein A, which is a transcription activator. The 5****G allele could be incorporated with protein B, which hinders the combination of the transcription activator. The action locus is certified near the alleles. The 4****G homozygote lacks the combination site with B factor, thus the 4****G homozygote could not be combined with protein B, which contributes to elevated plasma PAI-1 activity [23], [24].

The relationship between *PAI-1* 4G/5G gene polymorphism and CAD risk is subject to much controversy worldwide. In 2003, Böttiger C et al. reported that 4G/5G polymorphism of the *PAI-1* gene is not associated with an increased risk of thrombotic and restenotic events after coronary artery stenting in Germany [25]. In 2007, Sampaio MF et al. reported that acute myocardial infarction (AMI) is not associated with *PAI-1* gene polymorphisms in young adults in Brazil [26]. By contrast, Juhan-Vague I et al. found that PAI-1 played a role in MI risk in the presence of underlying insulin resistance. A significant interaction between insulin or proinsulin and –675****4G/5G polymorphism is observed in MI risk in France [27]. In 2008, Onalan O et al. reported that the *PAI-1* 4G/4G genotype is an independent predictor for the development of MI in a Turkish population [28].

The present research has some limitations. Large-scale studies on the relationship between *PAI-1* 4G/5G gene polymorphism and CAD are still inadequate. *PAI-1* is influenced not only by *PAI-1* 4G/5G gene polymorphism, but also by environmental factors, such as the concentration of blood sugar, insulin, triglycerides, and so on [29]. Elevated plasma PAI-1 activity is also associated with the progress of CAD. Plasma PAI-1 activity in the acute coronary syndrome is much higher than that in stable CAD patients, suggesting that PAI-1 plays a more important role in the acute phase of CAD than in the other phases [30].

The current meta-analysis indicated that the 4****G allele of *PAI-1* 4G/5G gene polymorphism might increase the CAD risk in the Chinese Han population. Therefore, significant potential guidance for formulating CAD individual therapies is provided. Taking into account the aforementioned limitations, further studies are highly needed in the future.

## Supporting Information

File S1 **PRISMA 2009 Checklist.**
(DOC)

File S2 **PRISMA 2009 Flow Diagram.**
(DOC)

## References

[1] Hamsten A, Wiman B, de Faire U, Blombäck M (1985). Increased plasma levels of a rapid inhibitor of tissue plasminogen activator in young survivors of myocardial infarction.. N Engl J Med.

[2] Held C, Hjemdahl P, Rehnqvist N, Wallén NH, Forslund L (1997). Haemostatic markers, inflammatory parameters and lipids in male and female patients in the Angina Prognosis Study in Stockholm (APSIS). A comparison with healthy controls.. J Intern Med.

[3] Eriksson P, Kallin B, van’t Hooft FM, Båvenholm P, Hamsten A (1995). Allele-specific increase in basal transcription of the *plasminogen-activator inhibitor 1* gene is associated with myocardial infarction.. Proc Natl Acad Sci U S A.

[4] Ye Z, Liu EH, Higgins JP, Keavney BD, Lowe GD (2006). Seven haemostatic gene polymorphisms in coronary disease: meta-analysis of 66,155 cases and 91,307 controls.. Lancet.

[5] Isordia-Salas I, Leaños-Miranda A, Sainz IM, Reyes-Maldonado E, Borrayo-Sánchez G (2009). Association of the *plasminogen activator inhibitor-1* gene 4G/5G polymorphism with ST elevation acute myocardial infarction in young patients.. Rev Esp Cardiol.

[6] Abboud N, Ghazouani L, Saidi S, Ben-Hadj-Khalifa S, Addad F (2010). Association of *PAI-1* 4G/5G and -844G/A gene polymorphisms and changes in PAI-1/tissue plasminogen activator levels in myocardial infarction: a case-control study.. Genet Test Mol Biomarkers.

[7] Rallidis LS, Gialeraki A, Merkouri E, Liakos G, Dagres N (2010). Reduced carriership of 4G allele of *plasminogen activator inhibitor-1* 4G/5G polymorphism in very young survivors of myocardial infarction.. J Thromb Thrombolysis.

[8] Ashavaid TF, Todur SP, Kondkar AA, Nair KG, Shalia KK (2011). Platelet polymorphisms: frequency distribution and association with coronary artery disease in an Indian population.. Platelets.

[9] Dai Y, Gao RL, Ye Y, Wu YJ, Chen JL (2001). The 4G/5G genetic polymorphism of the plasminogen activator inhibitor-1 (PAI-1) gene is not associated with plasma PAI-1 antigen level and the risk of coronary artery disease in Chinese.. Chinese Circulation Journal.

[10] Li XS, Xian SX, Huang HQ (2002). Relationship between plasminogen activator inhibitor-1 gene and coronary heart diseasewith blood-stagnation syndrome.. Journal of Guangzhou University of Traditional Chinese Medicine.

[11] Fu Y, Wang XD, Zhai YL, Fan Z, Yang L (2001). The 4G/5G polymorphism of the *plasminogen activator inhibitor-1* gene in patients with coronary heart disease.. Journal of Capital University of Medical Sciences.

[12] Guan L, Ji X, Wang J, Zhang A, Zhang Y (2002). Association of *plasminogen activator inhibitor-1* gene 4G/5G polymorphism and coronary heart disease in Chinese patients.. Zhonghua Yi Xue Yi Chuan Xue Za Zhi.

[13] Wang YS, Wang SY, Zhang M, Bai CX, Li SQ (2003). Polymorphisms of *PAI-1* 4G/5G in patients with coronary heart disease complicated with or without OSAHS.. New Medicine.

[14] Yin SQ, Liu S, Shun GY, Kan YD, Xie HQ (2003). Correlation between plasminogen activator inhibitor-1 and its gene polymorphism and acute myocardial infarction.. Tianjin Med J.

[15] Xia DS, Cao J, Song YQ, Hu SY, Guo QY (2006). Association between serotonin transporter and *plasminogen activator inhibitor-1* gene polymorphisms and depressive disorder in patients with coronary heart disease.. Chin J of Behavioral Med Sci.

[16] Zhan M, Zhou Y, Han Z (2003). *Plasminogen activator inhibitor-1* 4G/5G gene polymorphism in patients with myocardial or cerebrovascular infarction in Tianjin, China.. Chin Med J (Engl).

[17] Cochran WG (1968). The effectiveness of adjustment by subclassification in removing bias in observational studies.. Biometrics.

[18] Mantel N, Haenszel W (1959). Statistical aspects of the analysis of data from retrospective studies of disease.. J Natl Cancer Inst.

[19] DerSimonian R, Laird N (1986). Meta-analysis in clinical trials.. Control Clin Trials.

[20] Egger M, Davey Smith G, Schneider M, Schneider M, Minder C (1997). Bias in meta-analysis detected by a simple, graphical test.. British Medical Journal.

[21] Binder BR, Christ G, Gruber F, Grubic N, Hufnagl P (2002). Plasminogen activator inhibitor 1: physiological and pathophysiological roles.. News Physiol Sci.

[22] Franco RF, Reitsma PH (2001). Gene polymorphisms of the haemostatic system and the risk of arterial thrombotic disease.. Br J Haematol.

[23] Dawson SJ, Wiman B, Hamsten A, Green F, Humphries S (1993). The two allele sequences of a common polymorphism in the promoter of the *plasminogen activator inhibitor-1* (*PAI-1*) gene respond differently to interleukin-1 in HepG2 cells.. J Biol Chem.

[24] Eriksson P, Kallin B, Van’t Hooft FM, Båvenholm P, Hamsten A (1995). Allele-specific increase in basal transcription of the *plasminogen-activator inhibitor 1* gene is associated with myocardial infarction.. Proc Natl Acad Sci USA.

[25] Böttiger C, Koch W, Lahn C, Mehilli J, Von Beckerath N (2003). 4G/5G polymorphism of the *plasminogen activator inhibitor-1* gene and risk of restenosis after coronary artery stenting.. Am Heart J.

[26] Sampaio MF, Hirata MH, Hirata RD, Antos FC, Picciotti R (2007). AMI is associated with polymorphisms in the NOS3 and FGB but not in *PAI-1* genes in young adults.. Clin Chim Acta.

[27] Juhan-Vague I, Morange PE, Frere C, Aillaud MF, Alessi MC (2003). The *plasminogen activator inhibitor-1* -675 4G/5G genotype influences the risk of myocardial infarction associated with elevated plasma proinsulin and insulin concentrations in men from Europe: the HIFMECH study.. J Thromb Haemost.

[28] Onalan O, Balta G, Oto A, Kabakci G, Tokgozoglu L (2008). *Plasminogen activator inhibitor-1* 4G4G genotype is associated with myocardial infarction but not with stable coronary artery disease.. J Thromb Thrombolysis.

[29] Dawson S, Hamsten A, Wiman B, Henney A, Humphries S (1991). Genetic variation at the plasminogen activator inhibitor-1 locus is associated with altered levels of plasma plasminogen activator inhibitor-1 activity.. Arterioscler Thromb.

[30] Iwai N, Shimoike H, Nakamura Y, Tamaki S, Kinoshita M (1998). The 4G/5G polymorphism of the *plasminogen activator inhibitor* gene is associated with the time course of progression to acute coronary syndromes.. Atherosclerosis.

