# Supplementary material for: Plasminogen Activator Inhibitor-1 4G/5G Gene Polymorphism and Coronary Artery Disease in the Chinese Han Population: A Meta-Analysis
Source: PLoS One. 2012 Apr 4;7(4):e33511. doi: 10.1371/journal.pone.0033511 (PMC3319560; doi:10.1371/journal.pone.0033511)
Supplement: File S2 — PRISMA 2009 Flow Diagram. (DOC) [file pone.0033511.s002.doc]

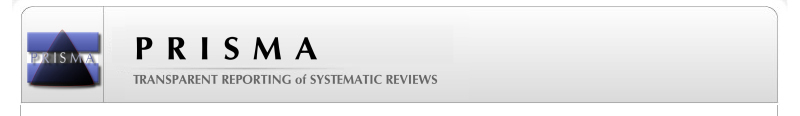
**PRISMA 2009 Flow Diagram**

**Screening**

**Included**

**Eligibility**

**Identification**

Records identified through database searching
(n = 22 )

Additional records identified through other sources
(n =0 )

Records after duplicates removed
(n =19)

Records screened
(n = 14 )

Records excluded for reviews (n=5)
(n = 15 )

Full-text articles assessed for eligibility
(n=10)

Full-text articles excluded, for no association with *PAI-1* 4G/5G gene polymorphism (n = 4 )
(n = )

Studies included in qualitative synthesis
(n = 8 )

Studies included in quantitative synthesis (meta-analysis)
(n =8 )

Records excluded for deviating from HWE (n=2)
(n = 15 )

Records excluded for redundant studies (n=3)
(n = 15 )
